# A comment on “Acute effects of a single bout of high-intensity strength and endurance exercise on cognitive biomarkers in young adults and elderly men: a within-subjects crossover study”

**DOI:** 10.1186/s12967-025-07204-9

**Published:** 2025-10-24

**Authors:** Jiaojiao Shen, Xiangyang Mei

**Affiliations:** 1 Center for Rehabilitation Medicine, Department of Anesthesiology, Zhejiang Provincial People’s Hospital (Affiliated People’s Hospital, Hangzhou Medical College), Hangzhou, Zhejiang China; 2 Research Institute of Anesthesiology and Perioperative Medicine, Hangzhou Medical College, Hangzhou, Zhejiang China

To Editor:

We have carefully reviewed the recent article “Acute effects of a single bout of high-intensity strength and endurance exercise on cognitive biomarkers in young adults and elderly men: a within-subjects crossover study” by Mosti et al. [1]. This study investigates serum concentrations of three neurocognitive biomarkers (i.e., Klotho, brain-derived neurotrophic factor (BDNF), and glycosylphosphatidylinositol-specific phospholipase D1 (GPLD1)) after acute strength and aerobic exercise, as well as skeletal muscle gene expression, and analyzes the possible influence of sex and age, which may have beneficial implications for the management of mental and neurocognitive impairments.

The authors discretely coded the variables group with three levels (i.e., young women, young men, elderly men) and time with four levels (i.e., t_1_–t_4_) to analysis serum levels at baseline and across time. However, the lack of an elderly female population makes it hard to assess comprehensively the impact of sex and age on cognitive biomarkers. The blood samples were collected by standard venipuncture at the following four time points: at baseline (i.e., before physical testing/exercise, pre, t_1_), immediately after (i.e., 0–5 min, post, t_2_), 3 h (t_3_) and 24 h (t_4_) after the acute exercise bouts. The results showed that serum Klotho and BDNF levels peaked immediately after exercise in all groups, while serum GPLD1 levels were highest at 3 h (young groups only). Given that blood sampling occurred only at t_2_ and t_3_, the prolonged interval may fail to capture transient biomarker peaks. Adding sampling time points between t_2_ and t_3_ (e.g., 30 min, 1 h, and 2 h post-exercise) would more accurately identify peak serum levels of each biomarker.

Muscle biopsies were obtained from a volunteer subgroup (*n* = 22). PCR analysis revealed increased skeletal muscle Klotho mRNA expression post-exercise, while BDNF and GPLD1 expression decreased. Including protein expression analyses in the biopsies would have been beneficial to interpret the muscle-specific response from exercise. Also, the sample size of muscle biopsy is relatively low (*n* = 22), which may compromise statistical power. Expanding the sample size, particularly for subgroup analysis, could enhance the reliability of the results.

The study did not conduct cognitive assessments before and after acute exercise, thus precluding direct correlation between biomarker dynamics and cognitive improvement. Moreover, as the cohort exclusively comprised healthy adults, the findings may not generalize to clinical populations (e.g., individuals with cognitive impairment) [2].

The analysis of acute exercise-induced changes in serum cognitive biomarkers appropriately accounted for key confounders including age and gender. However, diet and sleep may also modulate biomarker expression: evidence indicates high-fat diets reduce hippocampal BDNF expression in rats [3], while sleep duration negatively correlates with serum Klotho levels [4]. Incorporating diet and sleep as covariates would enhance the study’s comprehensiveness and reliability.

This study delivers substantive insights into exercise-induced effects on cognitive biomarkers, yet limitations persist in sample diversity, cognitive function correlation, temporal sampling design, and molecular mechanism validation. To advance scientific rigor, future research should expand participant cohorts, incorporate standardized cognitive assessments, and optimize experimental protocols. Implementing these refinements will enhance our understanding of exercise-brain health dynamics, providing critical foundations for developing targeted clinical interventions. Ultimately, such methodological advancements may yield novel therapeutic strategies for mental and cognitive disorders—potentially alleviating their growing global disease burden through evidence-based exercise prescriptions.

## Data Availability

Not applicable.
